# Sex-Dependent Psychoneuroendocrine Effects of THC and MDMA in an Animal Model of Adolescent Drug Consumption

**DOI:** 10.1371/journal.pone.0078386

**Published:** 2013-11-04

**Authors:** Alvaro Llorente-Berzal, Emma Puighermanal, Aurelijus Burokas, Andrés Ozaita, Rafael Maldonado, Eva M. Marco, Maria-Paz Viveros

**Affiliations:** 1 Departamento de Fisiología (Fisiología Animal II), Facultad de Biología, Universidad Complutense de Madrid, Madrid, Spain; 2 Instituto de Investigación Sanitaria del Hospital Clínico San Carlos, Madrid, Spain; 3 Laboratori de Neurofarmacologia, Departament de Ciències Experimentals i de Salut, Universitat Pompeu Fabra, Barcelona, Spain; Universidade do Estado do Rio de Janeiro, Brazil

## Abstract

Ecstasy is a drug that is usually consumed by young people at the weekends and frequently, in combination with cannabis. In the present study we have investigated the long-term effects of administering increasing doses of delta-9-tetrahydrocannabinol [THC; 2.5, 5, 10 mg/kg; i.p.] from postnatal day (pnd) 28 to 45, alone and/or in conjunction with 3,4-methylenedioxymethamphetamine [MDMA; two daily doses of 10 mg/kg every 5 days; s.c.] from pnd 30 to 45, in both male and female Wistar rats. When tested one day after the end of the pharmacological treatment (pnd 46), MDMA administration induced a reduction in directed exploration in the holeboard test and an increase in open-arm exploration in an elevated plus maze. In the long-term, cognitive functions in the novel object test were seen to be disrupted by THC administration to female but not male rats. In the prepulse inhibition test, MDMA-treated animals showed a decrease in prepulse inhibition at the most intense prepulse studied (80 dB), whereas in combination with THC it induced a similar decrease at 75 dB. THC decreased hippocampal Arc expression in both sexes, while in the frontal cortex this reduction was only evident in females. MDMA induced a reduction in ERK1/2 immunoreactivity in the frontal cortex of male but not female animals, and THC decreased prepro-orexin mRNA levels in the hypothalamus of males, although this effect was prevented when the animals also received MDMA. The results presented indicate that adolescent exposure to THC and/or MDMA induces long-term, sex-dependent psychophysiological alterations and they reveal functional interactions between the two drugs.

## Introduction

MDMA (3,4-methylenedioxymethamphetamine) is a psychostimulant drug commonly known as ecstasy. This drug induces rapid release of serotonin and inhibition of its reuptake, as well as affecting other neurotransmitters, such as dopamine and norepinephrine [1], [2]. It also produces a marked increase in metabolic activity, free radical production and oxidative stress [3]. The acute effects of MDMA last for 3 to 5 hours in humans, and they include euphoria, relaxation, an increase in sociability, empathy and energy [4], [5]. Ecstasy users also show other physiological modifications, such as body temperature deregulation and weight loss [6], [7]. In the long-term, heavy MDMA use can lead to mood and cognitive problems, including impulsivity, memory deficits, anxiety and depression [3], [8], [9]. Ecstasy is a widely used recreational drug that is commonly consumed by teenagers and young adults [10] who self-administer the drug at raves and dance club venues [3]. Most ecstasy users are polydrug users who consume MDMA at weekends in combination with other drugs of abuse, including cannabis [11], [12].

Cannabis is the illicit drug most commonly used by adolescents and young adults, and its main psychoactive compound is delta-9-tetrahydrocannabinol (THC) [13], [14]. THC mainly exerts its psychoactive effects though the activation of CB1 cannabinoid receptors primarily located in the central nervous system. CB1 receptors are most prominent during adolescence [15] when important rearrangements of neurotransmitter systems occur. Indeed, the chronic activation of CB1 receptors during adolescence has been reported to induce cognitive impairments and emotional alterations in adulthood, these resembling psychotic-like symptoms in both humans and animal models [16]–[20]. Therefore, cannabis consumption during this critical period, i.e. adolescence, could exert profound effects on the maturation and normal function of the circuits in the developing brain, the deleterious effects of which would be noticeable in later life [13], [14].

Polydrug use among young people is a very frequent phenomenon and it has increased in recent years. In particular, cannabis is the most widely consumed illegal co-drug in MDMA users, especially among younger adults [21], [22]. In a recent study of college students from the East Coast of the United States, 98% of ecstasy users had also consumed cannabis [23]. The motivation for polydrug use might be influenced by psychophysiological aspects, since decreasing concentrations of MDMA in the brain can lead to dysphoric symptoms including anxiety, agitation, insomnia and depression. Cannabis use might be considered as an attempt to relieve the negative symptoms associated with the “come-down” from ecstasy (see [21] and [22] for review). Whatsoever, the frequent combination of THC and MDMA by young people is a cause of concern, and the impact of this combination on later adult life deserves further study.

Previous studies have focused on the neurochemical and behavioral effects of the acute co-administration of THC and MDMA in adult rodents [24]–[26]. However, research on chronic exposure to this drug combination is very scarce and to the best of our knowledge, the only study published on the effects of the chronic exposure to MDMA and THC during adolescence refers only to male rats [27]. Sex-differences affect multiple psychobiological aspects of addiction [28]–[31] and indeed, clinical studies have shown that females are more susceptible to the effects of drugs of abuse than males [31]. We have extensively studied sexual dimorphisms after chronic cannabinoid treatment during adolescence [15]–[17], [20], [28]. However, only a few studies have focused on sexual dimorphism of adults in response to MDMA [7], [32], [33] and not on adolescent animals.

On the basis of our earlier findings, we designed an experimental protocol in which adolescent rats of both sexes were treated chronically with increasing doses of THC [34] and/or MDMA following a weekend consumption schedule [35]. Body temperature was evaluated after the first and last day of MDMA administration and one day after the end of the pharmacological treatment, animals were tested in the holeboard test as an independent measure of motor activity and site-directed exploration, and in the elevated plus maze for the evaluation of anxiety-like responses [36]. As adults, the animals were also tested in the novel object recognition test to assess working memory [37], and with the prepulse inhibition of acoustic startle to evaluate attention abilities [38]. In order to correlate these behavioral responses with specific changes in intracellular signaling that may be promoted by THC and MDMA, we also measured the expression of the extracellular signal-regulated kinases 1 and 2 (ERK1/2), as well as that of the activity-regulated cytoskeletal-associated protein (Arc), both in the hippocampus and prefrontal cortex of these animals. ERK1/2 is believed to be modulated in the rat brain by chronic administration of THC [39] and MDMA [40], and these modifications have been related to changes in mood and anxiety-like behavior [41]. Similarly, Arc mRNA expression was modified by acute MDMA administration in the cortical and hippocampal regions of the rat [2], and this immediate early gene is translated to a cytosolic protein involved in synaptic plasticity, long-term potentiation and learning [42]. In addition, body weight was registered throughout the study, as were circulating levels of leptin and corticosterone, two hormones that respond to both cannabinoids [15], [43] and MDMA [44], [45]. Finally, prepro-orexin mRNA expression was analyzed in the hypothalamus since its expression is modulated by feeding [46] and these two drugs modify food intake.

## Materials and Methods

All the experiments performed here were approved by the Comité de Experimentación Animal (CEA) de la Universidad Complutense, and they were designed and performed in compliance with the Royal Decree 1201/2005, October 21, 2005 (BOE n° 252) regarding the protection of experimental animals and the European Communities Council Directive of 24 November 1986 (86/609/EEC).

### Animals

*Wistar* albino rats of both sexes were used in this study. Experiments were carried out on the offspring of rats purchased from Harlan Laboratories (Milan, Italy), which were mated (one male×two females for ten consecutive days) in our animal facilities approximately 2 weeks after their arrival. After mating, female rats were housed individually and the animals were monitored daily for parturition. On the day of birth, postnatal day (pnd) 0, the litters were adjusted and sex balanced to eight pups per dam (four males and four females). At weaning (pnd 22), male and female rats were housed separately as pairs of sibling animals. A total of 128 male and female animals from 16 litters were used in the experiments. All the animals were maintained under constant conditions of temperature (22±2°C) and humidity (50±2%) on a reverse 12 h dark/light cycle (lights on at 20:00), with free access to food (commercial diet for rodents A04/A03; Safe, Augy, France) and water. It is worth mentioning that the stage of the estrous cycle was not examined in these studies. Given the length of the experimental protocol, we preferred not to perform periodic vaginal smears that would represent an additional source of stress to our animals and thus, might become a confounding factor in our study.

### Drug treatments

Delta-9-tetrahydrocannabinol (THC, commercialized as dronabinol) was purchased from THC Pharm GmbH (Frankfurt, Germany) and dispersed in ethanol, cremophor (Sigma-Aldrich, Spain) and saline (1∶1∶18) as described previously [15], [47]. 3,4-methylenedioxymethamphetamine (MDMA) hydrochloride was purchased from Lipomed (Arlesheim, Switzerland) and solutions were prepared daily in saline (0.9% NaCl).

**Figure 1:**
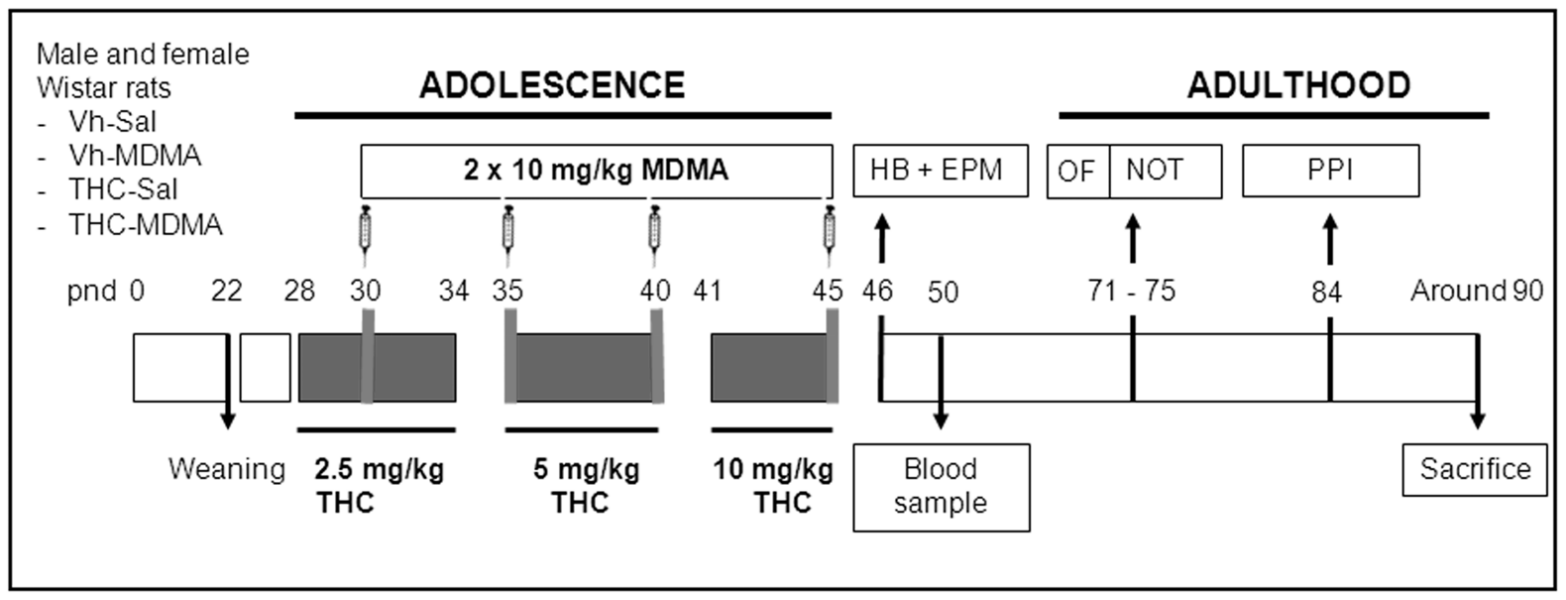
Timeline of the experimental design. Animals were exposed to increasing doses of THC (2.5, 5 and 10 mg/kg or vehicle, i.p.) from pnd 28 to 45, and to MDMA (10 mg/kg or saline, s.c.) twice daily every 5 days from pnd 30 to 45 with an inter-dose interval of 4 h (n = 16 animals per experimental group, see text for details). One day after the last drug administration, on pnd 46, the holeboard test (HB) was performed, immediately followed by the elevated plus maze test (EPM). The novel object test (NOT) was performed on pnd 75, and open-field (OF) data obtained from the first day of habituation to the arena on pnd 71 was also used. The animals were submitted to the prepulse inhibition test (PPI) on pnd 84. Thereafter, around pnd 90 (pnd 89–92), the animals were sacrificed and their brain was collected for future analyses. In addition, blood samples were extracted from the tail vein on pnd 50 for endocrine measurements, and from the trunk at sacrifice.

Drug treatments were administered only during adolescence, from pnd 28 to pnd 45 [48]. During this period, rats received intraperitoneal injections of increasing doses of THC (2.5 mg/kg from pnd 28 to 34; 5 mg/kg from pnd 35 to 40; 10 mg/kg from pnd 41 to 45) or of the vehicle alone, according to a protocol slightly modified from [34]. Additionally, every five days, from pnd 30, the animals received two daily injections of MDMA (10 mg/kg, s.c., calculated as the salt) or saline (Sal), with an inter-dose interval of 4 h (at approximately 10.00 and 14.00). This follows a protocol modified from Meyer et al. (2008) where the authors reported similarities between the model and the effects of regular weekend ecstasy consumption [35]. Both drugs were administered at a volume of 2 ml/kg (see Fig. 1 for details).

### Body weight control

Body weight (BW) was registered daily during the administration period, from pnd 28 to 45, and every five days thereafter, from pnd 50 to 70. The evolution of BW was calculated as the difference in BW in relation to the reference value at pnd 30. Two periods were considered for statistical analyses: during drug administration, adolescence; and as young adults once drug administration was withdrawn (from pnd 50 to 70).

### Body temperature measurements

Body temperature was measured by placing an electronic thermocouple rectal probe RTM-1 (Cibertec S.A., Madrid, Spain) in the rectum for 10 s. Rectal body temperature was measured on the first (pnd 30) and the last day (pnd 45) of MDMA (or Sal) administration. Body temperature was measured just before first MDMA (or Sal) injection, for baseline measures, and then, one and two hours later. Four hours later, immediately before the second MDMA (or saline) injection, body temperature was again registered, as well as one hour after.

### Behavioral testing

#### Holeboard

On pnd 46, animals were placed in the testing room for a 30 min. habituation period. The holeboard consisted in a squared arena (60 cm×60 cm×45 cm) with matte-painted metallic walls, and a plastic-covered wooden floor divided into 36 equal squares (10 cm×10 cm) and bearing four equally distant holes (3.8 cm in diameter). Animals were placed in the peripheral area of the arena for 5 min. under red light conditions and the animals' behavior was video recorded (Sony DCR-DVD310E) for subsequent evaluation. Rearing frequency (number of times that the animal stood on its rear limbs, vertical activity), and the frequency and duration of head-dipping exploration were recorded (RCV, Cibertec, Madrid, Spain). Locomotor activity in each region of the arena was measured by video tracking the animals (SMART version 2.5.20, Panlab, S.L.U, Barcelona, Spain) and the percentage of internal ambulation was calculated as an index of emotionality. Three animals accidentally fell out of the arena through one of the holes and were thus excluded from the analysis of the holeboard data. Nonetheless, these animals were submitted to the elevated plus maze in order to match the animals' conditions, although their behavior was not considered in the corresponding statistical analysis. The apparatus was carefully cleaned between tests on different animals with a 20% (v/v) ethanol solution.

#### Elevated plus-maze

The elevated plus-maze (EPM) is formed by two open arms (50 cm×10 cm) and two equally sized enclosed arms with 40 cm high walls, arranged so that the arms of the same type are opposite one another. The junction of the four arms formed a central square area (10 cm×10 cm). The apparatus was made of opaque black polyvinyl chloride (PVC) elevated to a height of 62 cm. On pnd 46, immediately after exposure to the holeboard, the animals were placed in the central platform of the apparatus facing one of the enclosed arms, and they were allowed to freely explore the maze for 5 min. under conditions of dim red light. Whenever an animal entered an arm with all four limbs, it was considered a visit, and the frequency and duration of the visits to the open and closed arm were recorded. Some animals fell from the plus-maze and were excluded from the analysis. Open arm entries and the time spent in the open arms was calculated as a percentage of the total entries into the arms and the total time spent in both arms, respectively, since these are considered the most relevant parameters related to anxiety in this test. By contrast, total arm entries were considered as an index of general motor activity [36]. The apparatus was carefully cleaned between tests on different animals with a 20% (v/v) ethanol solution.

#### Novel object test

The novel object test (NOT) was performed in a square arena (60 cm×60 cm×45 cm) with matte-painted metallic walls and a plastic-covered wooden floor divided by white painted lines into 36 squares (10 cm×10 cm). The test was performed as described previously by [37], with some minor modifications [16]. Animals were allowed to freely explore the arena for 5 min. under dim light conditions on 5 consecutive days (habituation period). On the first day of habituation (pnd 70), the arena was used as an open field and the animals' general activity was video recorded for subsequent behavioral evaluation (RCV, Cibertec, Madrid, Spain) and its locomotor activity was analyzed by video tracking (SMART, Panlab, S.L.U, Barcelona, Spain). Total ambulation and rearing frequency were registered, and the percentage of time spent in the internal area of the arena was calculated by dividing the time spent in the interior of the arena by the total duration of the test (5 min.), and then multiplying the resulting fraction by 100. On the test day (pnd 75), the rats were first exposed to two identical objects (two plastic boxes) until they explored the objects for 30 s or for a maximum period of 4 min., *training session.* Following a 4 h inter-trial interval, the rats were exposed to one of the previously encountered objects (familiar object, F1 or F2) and to a novel, unfamiliar object (metallic colored box, N) for 3 min., *test session*. The objects were not bigger than twice the size of a rat and they were located in contiguous corners, at a distance of 10 cm from the walls. At the beginning of each session, the animals were placed in the center of the apparatus facing the wall opposite to the objects. For each animal, the position of the objects was not changed between the training and the test session. However, the objects' position was changed between animals in order to avoid spatial preference. The apparatus and the objects were carefully cleaned between tests on different animals with a 20% (v/v) ethanol solution. Both training and test sessions were video recorded (Sony DCR-DVD310E) and the animals' behavior was later evaluated by an experienced observer by means of event-recorder software (RCV, Cibertec, Madrid, Spain). The time spent exploring the objects during the two sessions was registered, whereby exploration of an object was considered whenever animals pointed their nose toward an object at a distance ≤1 cm, while turning around, climbing and/or biting the objects was not considered as exploration [16], [37]. In the test session, the discrimination index was calculated as the difference between the time spent exploring the novel object (N) and the familiar one (F1 or F2) in relation to the total time spent exploring the objects [(N−F)/(N+F)]. Negative discrimination index values were transformed to zero since we assumed that animals exploring the familiar object longer than the novel one were not able to discriminate between the objects. Animals that explored for less than 30 s during the training session and those exclusively exploring only one of the objects during the test session were excluded from the statistical analyses.

#### Prepulse Inhibition

The startle device consisted of a non-restrictive Plexiglas cage (28 cm×16 cm×variable height) which encloses the sensor's platform but does not touch it. If the animal moves up or down, a transient force is developed on the platform and this transient force is measured at its peak, which represents the measure of the amplitude of the startle response. The rat's startle movements were transduced using an accelerometer and the data were monitored through a computer by using the MONRS v2.0 software (Cibertec S.A., Madrid, Spain). The startle response was recorded over 100 ms immediately following the pulse, with the startle device located in a constantly illuminated, sound-attenuating chamber (56 cm×14 cm×58 cm) and with a loudspeaker located in the top of the chamber.

On pnd 84, rats were placed in the startle chamber for a 5 min. habituation with 65 dB white background noise. The rats were then subjected to 10 startle trials (120 dB, 20 ms in duration), while for the prepulse inhibition (PPI) measurements animals were exposed to 10 blocks of five trials during which they were exposed to: 1) no startle stimulus (baseline activity, 65 dB); 2) the startle stimulus alone (120 dB, 20 ms. in duration); and 3) a startle stimulus occurring 100 ms. after the auditory prepulse (73, 75 or 80 dB, 20 ms. in duration). The startle stimuli were presented at a variable inter-trial interval of 10 to 20 s, which occurred in a semi-random manner with the restriction that each trial type had to occur in every five trial blocks. The cage was cleaned with a 20% ethanol solution between tests on different animals. The amount of PPI is expressed as the relative decrease in the amplitude of the startle response caused by the presentation of a prepulse and it was calculated according to the formula: 100×[(startle stimulus reactivity - startle stimulus reactivity in the presence of a prepulse)/startle stimulus reactivity] [38]. Animals that responded less to the startle stimulus alone than to the no startle stimulus in more than one block during the test session were excluded from the analyses.

### Plasma endocrine measurements

Blood samples were collected from the tail vein shortly after drug withdrawal, on pnd 50, into ice-cold EDTA capillary tubes (Microvette CB300, Sarsted, Granollers, Spain), and from the trunk at sacrifice (pnd 89–92), in vacuum blood collection EDTA tubes (Vacutest Plast, Kima, Arzergrande, Italy). The blood samples were centrifuged (3,000 rpm, 15 min. at 4°C), the plasma obtained and stored at −30°C until hormones were measured.

Corticosterone was measured using a solid phase ^125^I radioimmunoassay (ImmuchemTM Corticosterone kit, MP Biomedicals, Orangeburg, NY, USA) with a detection limit of 7.7 ng/ml, and intra-assay and inter-assay coefficients of variation less than 10%. Leptin was measured with an ELISA kit (B-Bridge International, Inc. Mountain View, CA, USA) according to the manufacturer's instructions. The assay sensitivity for the leptin assay was 0.5 ng/ml, with an inter-assay variation 6.5% and intra-assay variation of 3.7%. Absorbance in each well was measured using a microplate reader (Tecan Infinite M200 (Grödig, Austria) and the plasma concentrations calculated from the standard curve. All samples were run in duplicate and plasma hormone concentrations were calculated from the standard curve.

### Brain processing and analysis

At adulthood (pnd 89–92), animals were sacrificed by decapitation and their brain was rapidly extracted and dissected on ice. The frontal cortex, hippocampus and hypothalamus were obtained and stored at −80°C.

#### Immunoblotting of the frontal cortex and hippocampus

Protein samples were prepared as described previously [49] and they were analyzed in western blots probed with antibodies against ERK1/2 and phospho-ERK1/2 (Thr202/Tyr204: Sigma, Madrid, Spain), against Arc (Cell Signaling Technologies, Beverly, MA, USA) and against glyceraldehyde 3-phosphate dehydrogenase (GAPDH: Santa Cruz Biotechnologies, Santa Cruz, CA, USA). The blots were re-probed with GAPDH to ensure accurate protein loading. Optical densities of relevant immunoreactive bands were quantified after acquisition on a ChemiDoc XRS System (Bio-Rad, Hercules, CA, USA) controlled by the Quantity One software version 4.6.3 (Bio-Rad, Hercules, CA, USA). The data were expressed as the relative change in optical density with respect to the male or female control group, considered as 100%. Since samples from male and female animals were analyzed in separate blots, sex-dependent effects could not be analyzed for these measurements.

#### Quantitative real-time PCR analysis of the hypothalamus

Total RNA was isolated using the RNeasy Mini Kit (Qiagen® GmbH, Hilden, Germany), following the manufacturer's instructions, and the quality of the total RNA was assessed through the spectrophotometric ratio A260/A280 (1.9 to 2.1), while the total RNA concentration was measured using a NanoDrop™ spectrophotometer (Thermo Fisher Scientific, Wilmington, DE, USA). Reverse transcription was performed with 0.5 µg of total RNA from each animal to produce complementary DNA (cDNA) in a 20 µl reaction with 200 units of SuperScript III Reverse Transcriptase (Invitrogen, Carlsbad, CA, USA) and 500 ng oligo(dT)15 primers. The cDNA obtained was diluted 1∶10 and stored at −20°C for later use. The reactions were carried out at 25°C for 10 min, then 50 min at 42°C and 15 min at 70°C.

Real-time PCR was carried out on a ABI PRISM® 7700 Sequence Detection System (PE Applied Biosystems, Paisley, UK) using the SYBR Green PCR Master Mix (PE Applied Biosystems, Paisley, UK), according to the manufacturer's protocol. Briefly, 4 µl of the diluted cDNA template was amplified with SYBR Green in a 10 µl reaction containing 2× SYBR Green PCR Master Mix, 0.5 µM of each primer and de-ionized water. The reactions were incubated at 50°C for 2 min to activate uracil N-glycosylase, and then for 10 min at 95°C to inactivate this enzyme and activate the Amplitaq Gold polymerase. Subsequently, the reaction was subjected to 40 amplification cycles with a 15 s denaturation at 95°C, 1 min. annealing at 60°C, a 15 s extension at 95°C and a final cooling step to 60°C. The quantities of specific mRNA in the sample were measured according to the corresponding gene-specific standard curve. All the samples were tested in triplicate and the relative expression values were normalized to the expression of GAPDH.

Primers specific for rat prepro-orexin (GenBank ID: NM_013179; sense, 5′-ACCACTGCACCGAAGATACCA-3′; antisense, 5′-GGGAAAGTTAGGACTAGGA-3′) and rat GAPDH (NM_017008; sense, 5′-GCCAGCCTCGTCTCATAGACA-3′; antisense, 5′-GTCCGATACGGCCAAATCC-3′) were used, and the amplified products were separated on a 2% agarose gel and stained with ethidium bromide to confirm the specificity of the primers.

The quantities of specific mRNA in the sample were measured according to the corresponding gene-specific standard curve. All the samples were tested in triplicate and the relative expression values were normalized to the expression of GAPDH. Samples were analyzed by the double delta CT (ΔΔCT) method and the ΔΔCT values were calculated as the ΔCT of each test sample (different pharmacological treatments) minus the mean ΔCT of the calibrator samples (male or female Vh-Sal – control – group) for the prepro-orexin gene. The fold change was calculated using the equation 2^(−ΔΔCT)^. Since the samples from male and female animals were assayed separately, sex-dependent effects could not be analyzed for this parameter.

### Statistical analysis

Behavioral, physiological and endocrine data were analyzed by a three-way analysis of variance (ANOVA) with the factors sex (males *vs*. females), cannabinoid treatment (Vh *vs*. THC) and intermittent MDMA administration (Sal *vs*. MDMA). Due to the analytical limitations indicated above, the immunoblotting and PCR data were analyzed by two-way ANOVAs split by sex. Repeated measures ANOVAs were employed to analyze of the gain in body weight and temperature measurements. In the case of sphericity violation, a Greenhouse-Geisser correction was chosen to study the within-subjects factor effects. Additional two-way ANOVAs split by one of the independent factors (sex or pharmacological treatment) were performed to further clarify the results obtained. Normality and homocedasticity were assessed with Kolmogorov-Smirnov and Levene tests, respectively. When necessary, the data were transformed to achieve a normal distribution and when a normal distribution could not be achieved by transforming the data, the Kruskal-Wallis non-parametric test was performed. The frequency of events (i.e. deaths and falls from the EPM) was analyzed with a chi-squared test. Post hoc comparisons using the Tukey test were performed with a level of significance set at p<0.05. All statistical analyses were carried out with the SPSS 19.0 software package (SPSS Inc., Chicago, IL, USA).

## Results

### Body Weight gain

**Figure 2:**
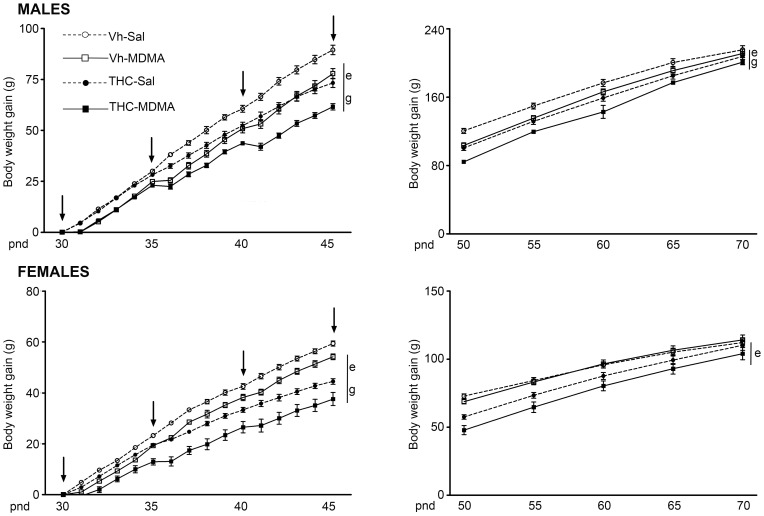
Evolution of body weight gain during the drug administration period (left panels) and following drug withdrawal (right panels). The animals were exposed to increasing doses of THC (2.5, 5 and 10 mg/kg or vehicle) from pnd 28 to 45, and to MDMA (10 mg/kg or saline) twice daily every 5 days from pnd 30 to 45 (see text for details). Body weight was registered daily during the administration period, from pnd 28 to 45, and every five days from pnd 50 to 70 (arrows indicate MDMA/Sal injections). The gain in body weight (in grams, g; mean ± S.E.M.) is expressed as the difference in body weight in males (upper panels) and females (lower panels) on each experimental day, and pnd 30 was established as the reference day. Number of animals per experimental group: males, Vh-Sal (16), Vh-MDMA (14), THC-Sal (16), THC-MDMA (14); and females, Vh-Sal (16), Vh-MDMA (14), THC-Sal (16), THC-MDMA (12). According to a repeated measures ANOVA (P<0.05): (e) significant overall effect of THC within the same sex group; (g) significant MDMA effect within the same sex group.

During drug treatment (adolescence) the rats gained significant body weight with age [F(2.5, 277.9) = 4579.31; p<0.001]. Significant sex differences were observed [F(1,110) = 276.55; p<0.001], with the body weight gain of males being higher than that of females, and there were significant effects of the two drugs employed, THC [F(1,110) = 102.58; p<0.001] and MDMA [F(1,110) = 98.78; p<0.001]. Moreover, a significant interaction between sex and MDMA administration was also detected [F(1,110) = 5.63; p<0.05]. Early during drug treatment (from pnd 31), animals administered MDMA showed a reduced rate in body weight gain that persisted until the last day of drug administration (pnd 45) in both males and females. In turn, THC administration during adolescence decreased the growth of male and female rats, although this effect was only evident from pnd 35 among males, while it was evident from pnd 31 in females. Both male and female adolescent animals treated with the combination of chronic THC and intermittent MDMA showed the lowest rate in body weight gain throughout the drug administration period (Fig. 2, left panels).

Following drug withdrawal, body weight gain still changed significantly with age [F(2.4, 262.2) = 2889.1; p<0.001]. In addition, significant effects of sex [F(1,110) = 1047.35; p<0.001] and the two drugs employed, THC [F(1,110) = 41.60; p<0.001] and MDMA [F(1,110) = 12.21; p<0.01], were still observed. Adolescent THC exposure induced a long-lasting reduction in body weight gain in male and female animals. However, this effect seemed to persist longer in males, an effect of THC on body weight gain was only observed in males at pnd 70 and not in females. Similarly, intermittent adolescent MDMA administration persistently reduced body weight in male animals (until pnd 65), whereas in females this drug seemed to decrease the gain in body weight only shortly after drug cessation (pnd 50), with this effect disappearing as these animals aged (Fig. 2, right panels).

It is worth mentioning that five animals died during the present experiment, all of which received MDMA (two males and three females), and two of which were chronically administered with the vehicle alone, while three chronically received increasing doses of THC. The frequency analysis of the death rate between saline (no deaths) and MDMA administered animals (5 out of 64) was 7.8%, reflecting a significant effect of this drug (χ^2^ = 5.20, p<0.05) in the absence of significant differences in the death rates depending upon sex or cannabinoid administration. Since the animals' rearing conditions changed when the cage partner died, the surviving sibling animal housed in the same cage was also removed from the statistical analyses.

### Body temperature measurement

**Figure 3:**
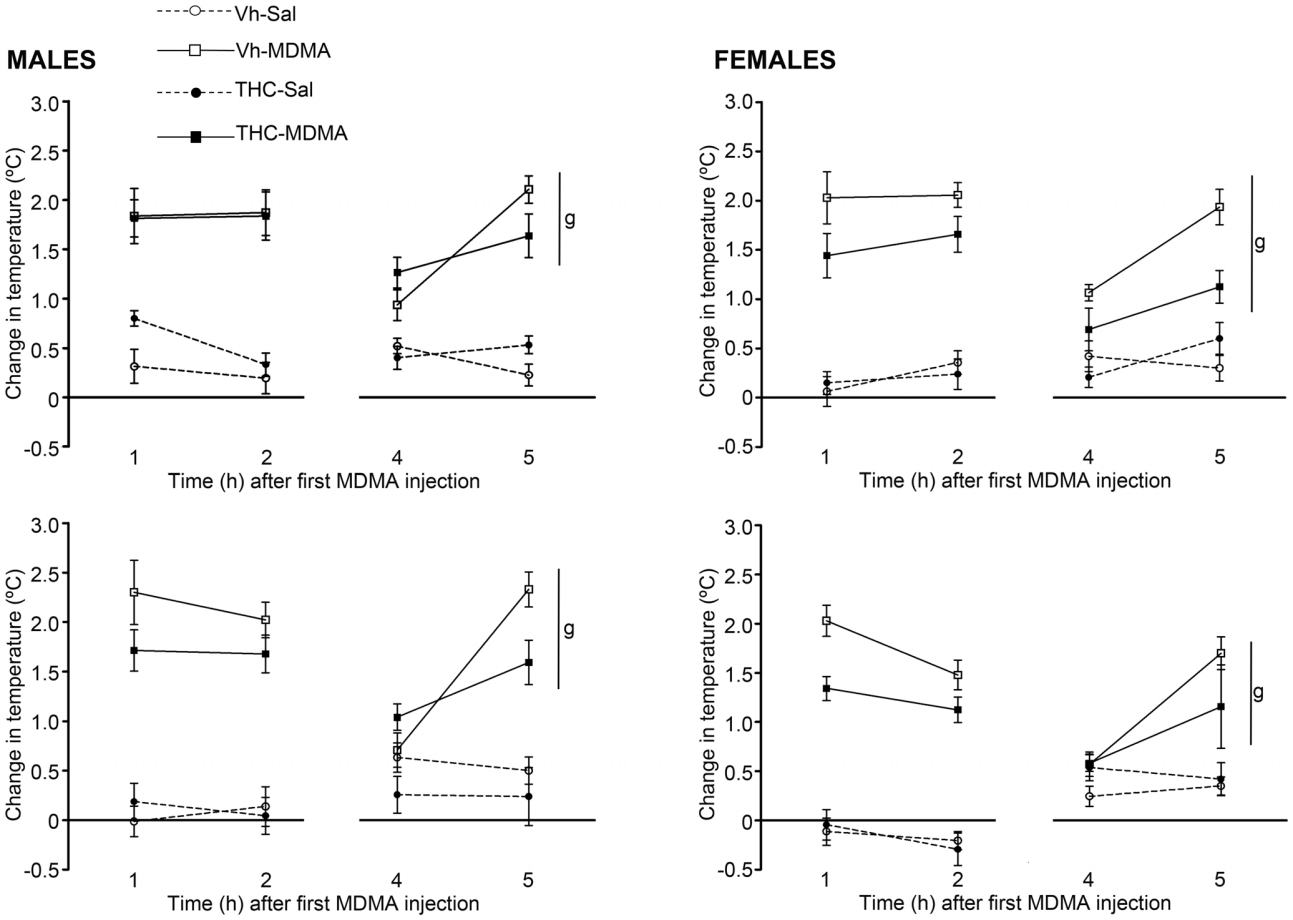
Changes in body temperature on the first (pnd 30) and last (pnd 45) days of MDMA administration. Animals were exposed to increasing doses of THC (2.5, 5 and 10 mg/kg or vehicle) from pnd 28 to 45 and to MDMA (10 mg/kg or saline) twice daily every 5 days from pnd 30 to 45 (see text for details). The animals' body temperature was measured just before the first MDMA (or Sal) injection to establish the baseline, and then one, two and four hours later, the latter immediately before the second MDMA (or Sal) injection, as well as one hour after that second injection (arrows indicate MDMA/Sal injections). The figures show the change in rectal temperature with respect to the baseline temperature (mean ± S.E.M.) expressed in degrees centigrade (°C). The number of animals per experimental group: males, Vh-Sal (16), Vh-MDMA (14), THC-Sal (16), THC-MDMA (14); and females, Vh-Sal (16), Vh-MDMA (14), THC-Sal (16), THC-MDMA (12). According to a repeated measures ANOVA (P<0.05), (g) represents a significant MDMA effect within the same sex group.

The rats' body temperature was analyzed by comparing the changes from the first (pnd 30) and last (pnd 45) days of MDMA administration (Fig. 3). Rectal temperature was measured just before the first MDMA (or Sal) injection (baseline), and then one, two and four hours later, as well as immediately before the second MDMA (or Sal) injection and one hour later. On the two days of body temperature measurement, rectal temperature changed significantly over the time-points selected for the measures [pnd 30, F(3.56,391.25) = 125.43; p<0.001 and pnd 45, F(3.29,360.87) = 74.62; p<0.001 respectively]. On the first day of MDMA administration, a baseline body temperature of 37.5±0.08°C and 37.8±0.08°C was recorded in male and female animals, respectively. On pnd 30, a significant overall effect of MDMA on body temperature was observed [F(1,110) = 188.29; p<0.001], together with a significant THC×MDMA interaction [F(1,110) = 5.11; p<0.05]. Further analyses revealed that MDMA significantly increased body temperature (i.e.: it caused hyperthermia) in both male and female adolescent animals [F(1,56) = 101.28; p<0.001 and F(1,54) = 87.48; p<0.001], whereas the significant THC×MDMA interaction was only evident among female animals [F(1,54) = 4.63; p<0.05]. Thus, THC administration seemed to counterbalance the MDMA-induced hyperthermia exclusively among females (Fig. 3, upper panels).

On pnd 45, significant overall effects of sex [F(1,110) = 8.13; p<0.01] and MDMA administration [F(1,110) = 169.82; p<0.001] were observed. Changes in body temperature were larger in males than females and MDMA administration induced a significant increase in body temperature (hyperthermia) on pnd 45, independently of the animal's sex, indicating no habituation to this hyperthermic effect (Fig. 3, lower panels).

### Behavioral testing

To facilitate the reading and comprehension of the behavioral results, the corresponding 3-way ANOVA data have been included in table 1.

**Table 1:** Main results from the statistical analysis of behavioral data.

Behav. test	Parameter analyzed	Statistics	Sex	THC	MDMA	Sex×THC	Sex×MDMA	THC×MDMA	Sex×THC×MDMA
HB	Total ambulation	F_(1,107)_	-	-	-	-	-	-	-
		p-value	-	-	-	-	-	-	-
	% internal ambulation	F_(1,107)_	-	4.35	-	-	-	-	-
		p-value	-	<0.05	-	-	-	-	-
	Head-dipping frequency	F_(1,107)_	-	-	7.64	-	-	-	-
		p-value	-	-	<0.01	-	-	-	-
	Head-dipping duration	F_(1,107)_	-	-	8.50	-	-	-	-
		p-value	-	-	<0.01	-	-	-	-
EPM	Closed arm entries	H_(1)_	-	-	4.97	-	-	-	-
		p-value	-	-	<0.05	-	-	-	-
	% open arm entries	F_(1,81)_	4.29	-	8.50	-	-	-	-
		p-value	<0.05	-	<0.01	-	-	-	-
	% time in open arms	F_(1,81)_	4.32	-	-	-	-	8.21	-
		p-value	<0.05	-	-	-	-	<0.01	-
OF	Total ambulation	F_(1,110)_	64.64	-	-	-	-	-	-
		p-value	<0.001	-	-	-	-	-	-
	Rearing frequency	F_(1,110)_	48.72	-	-	-	-	-	-
		p-value	<0.001	-	-	-	-	-	-
	Internal time	F_(1,110)_	-	4.54	-	-	-	4.74	-
		p-value	-	<0.05	-	-	-	<0.05	-
NOT	Discrimination index	F_(1,99)_	5.84	-	-	5.86	-	-	-
		p-value	<0.05	-	-	<0.05	-	-	-
	Object exploration	F_(1,99)_	-	-	6.46	-	-	-	-
		p-value	-	-	<0.05	-	-	-	-
PPI	% PPI 73 dB	F_(1,106)_	4.23	-	-	-	-	-	-
		p-value	<0.05	-	-	-	-	-	-
	% PPI 75 dB	F_(1,106)_	8.98	-	5.32	-	-	4.24	-
		p-value	<0.01	-	<0.05	-	-	<0.05	-
	% PPI 80 dB	F_(1,106)_	8.89	-	4.57	-	-	-	-
		p-value	<0.01	-	<0.05	-	-	-	-

Behavioral tests were carried out on animals that had been exposed in the adolescent period to increasing doses of THC (2.5, 5 and 10 mg/kg or vehicle) from pnd 28 to 45 and to MDMA (10 mg/kg or saline) twice daily every 5 days from pnd 30 to 45 (see text for details). Three-way ANOVA analyses were performed, except for on the closed arm entries, with factors being sex (males *vs.* females), neonatal manipulation (control *vs.* MD) and drug treatment (Sal *vs.* Coc). The main statistical values are shown: F, with degrees of freedom, or H (in the case of Kruskal-Wallis) for closed arm entries, and p-values. Non-significant results are not included (−). Behavioral tests: HB, holeboard test; EPM, elevated plus maze; OF, open field; NOT, novel object test; PPI, prepulse inhibition test.

#### Holeboard

Neither of the drugs tested here produced significant effects on the overall locomotor activity, i.e.: total ambulation (Table 1). However, THC produced a significant effect on the percentage ambulation in the central region of the arena (Table 1), such that it decreased the distance traveled in the central zone (Table 2). By contrast, hole-directed explorative behavior was notably affected by MDMA administration, and both the frequency and time spent exploring the holes of the arena were significantly lower in animals that were administered this drug (see Table 1 and 2).

**Table 2:** Locomotor activity and exploration in the holeboard test.

		Total ambulation (m)	Internal ambulation (%)	Head-dipping (frequency)	Head-dipping (duration, s)
**Males**	Vh-Sal (16)	23.85±1.31	19.4±2.6	16.8±1.7	29.4±3.4
	Vh-MDMA (14)	21.11±1.45	16.7±1.9	13.0±1.4^c^	23.5±3.0^c^
	THC-Sal (16)	22.73±1.21	15.2±2.2^b^	16.3±1.1	33.1±2.7
	THC-MDMA (13)	23.43±1.06	14.9±1.1^b^	14.2±1.4^c^	27.9±3.4^c^
**Females**	Vh-Sal (16)	25.95±1.23	15.7±1.6	17.6±1.3	34.2±3.2
	Vh-MDMA (13)	24.21±1.06	18.4±1.4	14.6±1.3^c^	26.4±3.3^c^
	THC-Sal (15)	23.38±0.79	16.0±1.3^b^	17.3±1.6	38.1±3.4
	THC-MDMA (12)	22.68±1.86	13.8±2.6^b^	14.3±2.2^c^	28.3±5.3^c^

Locomotor and explorative activity was registered in the holeboard test on pnd 46 for male and female animals administered with vehicle (Vh) or increasing doses of THC (2.5, 5 and 10 mg/kg) from pnd 28 to 45, and with saline (Sal) or MDMA (10 mg/kg twice daily) every 5 day from pnd 30 (until pnd 45). The data are expressed as the mean ± S.E.M. In parenthesis, the number of animals per experimental group. ANOVA (P<0.05),

b significant overall effect of THC;

c significant overall effect of MDMA treatment.

It is worth mentioning that the 3 animals that accidentally fell out of the arena through one of the holes came from different experimental groups: one male THC-MDMA, one female Vh-MDMA, and another female THC-Sal. Thus, this event seems not to be related to a specific drug treatment.

#### Elevated plus-maze

**Figure 4:**
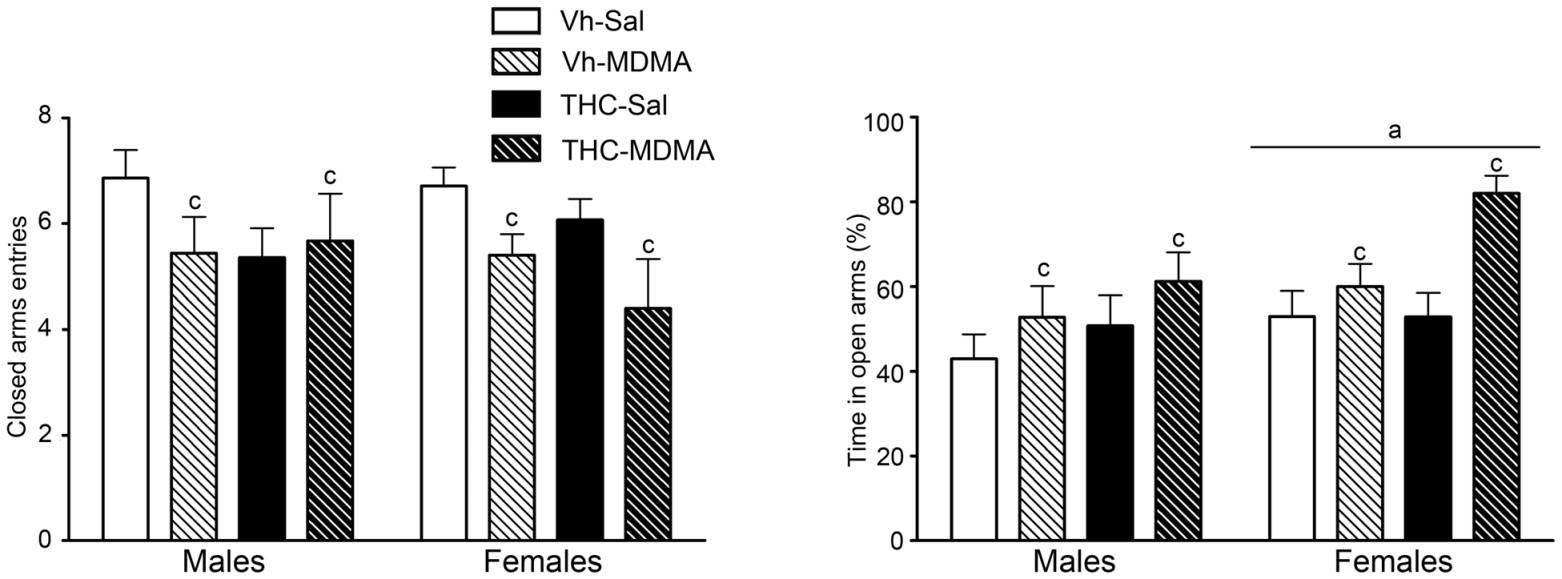
The elevated plus maze test was performed one day after the last drug administration, on pnd 46, immediately after the holeboard test. Animals were exposed to increasing doses of THC (2.5, 5 and 10 mg/kg or vehicle) from pnd 28 to 45, and twice daily to MDMA (10 mg/kg or saline) every 5 days from pnd 30 to 45 (see text for details). Histograms (mean ± S.E.M.) show the percentage time spent in the open arms (right panel) and the frequency of the closed arm entries (left panel). Number of animals per experimental group: males, Vh-Sal (14), Vh-MDMA (9), THC-Sal (14), THC-MDMA (9); and females, Vh-Sal (14), Vh-MDMA (10), THC-Sal (14), THC-MDMA (5). According to the Kruskal-Wallis test and ANOVA (P<0.05): (a) significant overall effect of sex; (c) significant overall effect of MDMA treatment.

MDMA had a significant effect on the closed arm entries when analyzed with the Kruskal-Wallis test (Table 1). MDMA reduced the number of visits to the closed arms, indicating a decrease in general locomotor activity. No significant differences were found in the number of open arm entries, although the ANOVA of the percentage of time spent in the open arms rendered a significant overall effect of sex and of MDMA. In order to confirm that this effect was independent from the reduced motor activity induced by the drug, we performed an additional analysis of the percentage of time spent in the open arms considering the closed arm entries as a covariate, confirming the effects of both factors [sex effect: F(1,80) = 4.06; p<0.05; and MDMA effect: F(1,80) = 4.99; p<0.05]. In summary, adolescent female rats explored the open arms of the maze for longer than the corresponding males, and MDMA administration increased the exploration time in the open-arms, independently of the MDMA-induced decrease in motor activity (Fig. 4).

It is worth mentioning that MDMA-treated animals more often fell from the EPM than saline-treated animals (χ^2^ = 10.53, p<0.01), with 19 out of 33 MDMA-treated animals falling from the EPM as opposed to 7 out of 56 saline treated animals. More specifically, the 26 animals that fell off the maze were distributed as follows: males: Vh-Sal (2), Vh-MDMA (5), THC-Sal (2), and THC-MDMA (4); females: Vh-Sal (2), Vh-MDMA (3), THC-Sal (1), and THC-MDMA (7).

#### Open field

In this test, a significant overall effect of sex was detected on total ambulation and frequency of rearing (Table 1), and as expected, females displayed greater motor activity than males (horizontal and vertical: see Table 3). In terms of the time spent exploring the interior of the arena, THC produced a significant overall effect, and there was a significant interaction between THC and MDMA (Table 1). THC administration decreased the time animals spent exploring the central region of the open field, indicative of increased emotionality/anxiety. MDMA induced a similar effect that was only evident in vehicle-treated animals [F(1,56) = 4.20; p<0.05], and not in THC-treated animals, possibly due to the low exploration of the internal zones of the arena exhibited by animals that had received THC (“floor effect”).

**Table 3:** General activity in an square arena.

		Total ambulation (m)	Rearing frequency	Internal time (%)
**Males**	Vh-Sal (16)	11.82±1.21	9.3±2.2	14.5±3.9
	Vh-MDMA (14)	9.33±1.12	7.1±1.3	8.7±3.3^d^
	THC-Sal (16)	11.87±1.24	11.2±2.9	6.1±2.6^b^
	THC-MDMA (14)	9.80±1.39	9.1±2.3	8.0±4.5^b^
**Females**	Vh-Sal (16)	18.86±1.62^a^	21.4±4.2^a^	14.5±4.4
	Vh-MDMA (14)	17.52±0.87^a^	22.6±2.3^a^	4.9±0.9^d^
	THC-Sal (16)	17.00±0.94^a^	20.1±3.2^a^	4.2±0.9^b^
	THC-MDMA (12)	17.71±1.23^a^	24.2±2.8^a^	7.1±1.7^b^

Locomotor and behavioral parameters registered on pnd 71 of male and female animals administered with vehicle (Vh) or increasing doses of THC (2.5, 5 and 10 mg/kg) from pnd 28 to 45, and with saline (Sal) or MDMA (10 mg/kg twice daily) every 5 day from pnd 30 to pnd 45. The data are expressed as the mean ± SEM. In parenthesis, the number of animals per experimental group. ANOVA (P<0.05),

a significant overall effect of sex;

b significant overall effect of THC;

d significant effect of MDMA in vehicle-treated animals.

#### Novel Object Test

**Figure 5:**
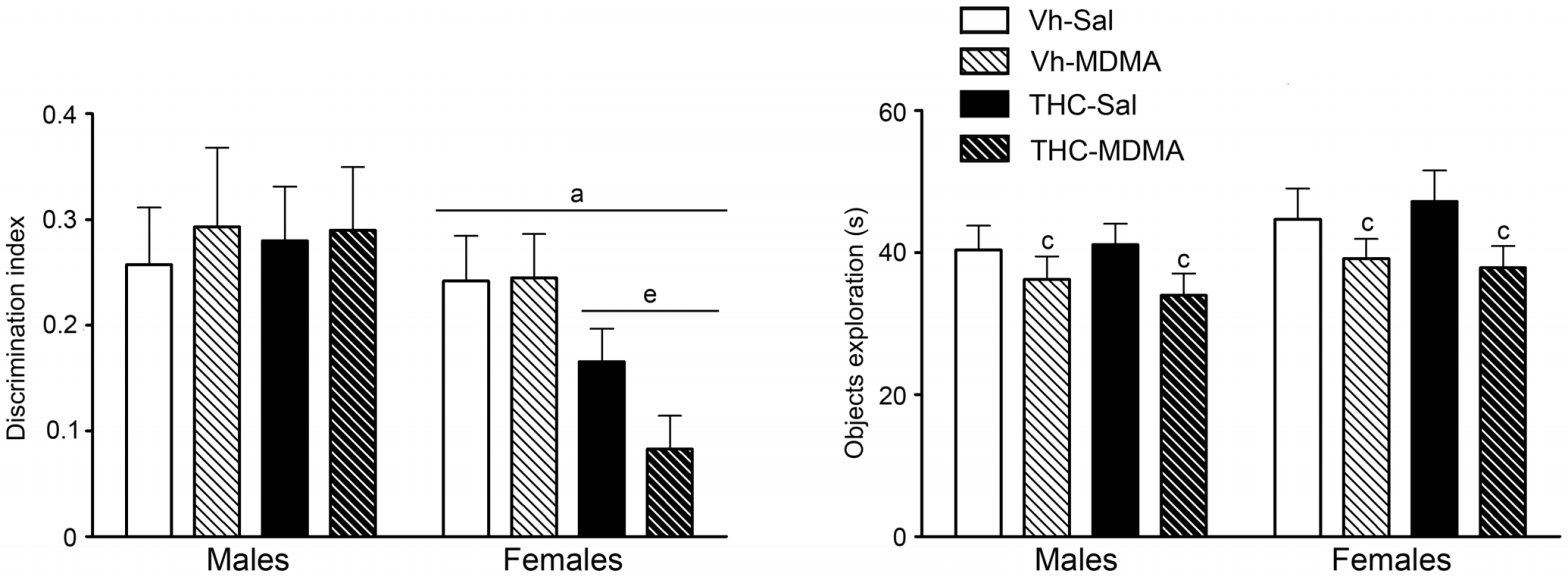
The novel object test (NOT) was performed on pnd 75. Animals were exposed to increasing doses of THC (2.5, 5 and 10 mg/kg or vehicle) from pnd 28 to 45 and to MDMA (10 mg/kg or saline) twice daily every 5 days from pnd 30 to 45 (see text for details). The histograms (mean ± S.E.M.) show the discrimination index (left panel) measured as the difference between the time spent exploring the novel object (N) and the time spent exploring the familiar one (F1 or F2) in function of the total time spent exploring the two objects during the test session [N−F/(N+F)]. The time animals spent exploring both objects during the test session is also shown (right panel). Number of animals per experimental group: males, Vh-Sal (15), Vh-MDMA (12), THC-Sal (15), THC-MDMA (11); and females, Vh-Sal (15), Vh-MDMA (13), THC-Sal (16), THC-MDMA (10). According to ANOVA (P<0.05): (a) significant overall effect of sex; (c) significant overall effect of MDMA treatment; (e) significant overall effect of THC within the same sex group.

In the novel object test, an analysis of the discrimination index revealed a significant overall effect of sex, together with a significant interaction between sex and THC (Table 1). Additional analyses revealed that adolescent THC administration exclusively affected female animals [F(1,50) = 9.08; p<0.01] and it did not modify this memory index in males. In addition, object exploration during the test session was significantly affected by the administration of MDMA, which decreased the time animals spent exploring both objects (see Table 1 and Fig. 5). As previously indicated, we excluded from the analyses any animals that explored the objects for less than 30 s in the training session, as well as those that only explored one of the objects in the test session. A total of 8 animals from the following experimental groups were disqualified based on these criteria: males from the Vh-Sal (1), Vh-MDMA (2), THC-Sal (1) and THC-MDMA (3) groups; females from the Vh-Sal (1), Vh-MDMA (1), THC-Sal (0) and THC-MDMA (2).

#### Prepulse inhibition test

**Figure 6:**
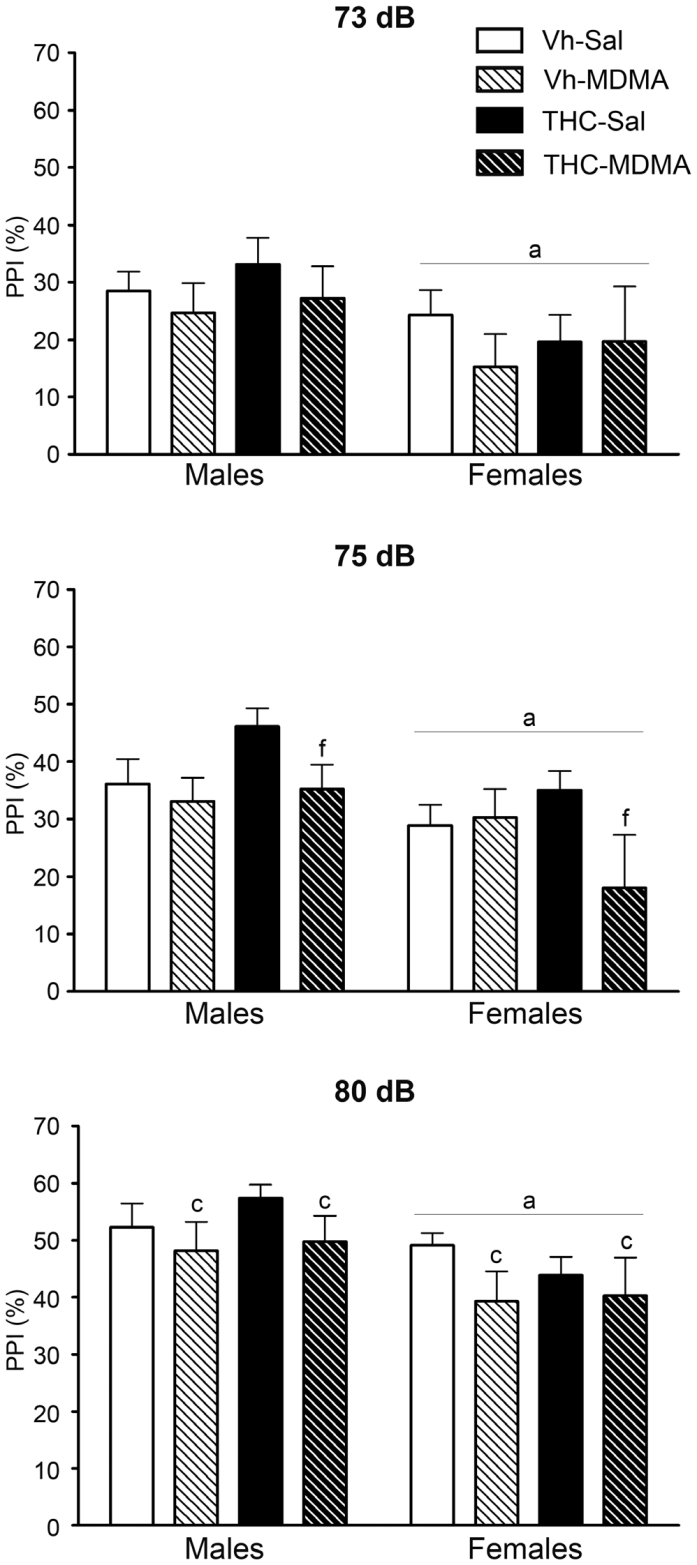
The prepulse inhibition test (PPI) was performed at adulthood, on pnd 84. Animals were exposed to increasing doses of THC (2.5, 5 and 10 mg/kg or vehicle) from pnd 28 to 45 and to MDMA (10 mg/kg or saline) twice daily every 5 days from pnd 30 to 45 (see text for details). The histograms represent the percentage of PPI (mean ± S.E.M.) with three different pre-pulse intensities (73, 75 and 80 dB). Number of animals per experimental group: males, Vh-Sal (16), Vh-MDMA (12), THC-Sal (16), THC-MDMA (14); and females, Vh-Sal (16), Vh-MDMA (14), THC-Sal (16), THC-MDMA (10). According to ANOVA (P<0.05): (a) significant overall effect of sex; (c) significant overall effect of MDMA treatment; (f) significant MDMA effect among THC-treated animals.

PPI responses to the three pre-pulse intensities were analyzed independently (Table 1) and for each pre-pulse intensity there was a significant overall effect of sex, suggesting that the sensorimotor gating was weaker in females than in males. A significant overall effect of MDMA was observed for 75 and 80 dB pre-pulse intensities and indeed, there was a significant interaction between THC and MDMA for the intermediate pre-pulse intensity (75 dB: Table 1). A more in depth analysis revealed that MDMA administration only reduced the percentage PPI response in animals that were also administered THC [F(1,52) = 8.63; p<0.01]. To summarize, adolescent MDMA administration significantly decreased the percentage of PPI at the highest pre-pulse intensity (80 dB), while following an intermediate pre-pulse stimulus (75 dB) this MDMA effect was only effective in reducing PPI response in combination with THC administration (Fig. 6). No significant effect of THC *per se* was found. Four animals (2 males Vh-MDMA and 2 females THC-MDMA) were excluded from this analysis since they responded less to the startle stimulus alone than to the no startle stimulus in more than one block during the test session.

### Hormone levels

#### Plasma corticosterone

When the corticosterone levels were analyzed by age, a marked sexual dimorphism for this endocrine parameter was evident at the two time points selected [pnd 50, F(1,62) = 66.99, p<0.001; and around pnd 90, F(1,62) = 62.11, p<0.001]. Plasma corticosterone levels were higher among females than males at both ages. While chronic THC administration during adolescence did not affect basal corticosterone levels in either sex, intermittent MDMA exposure significantly diminished long-term male corticosterone levels [F(1,32) = 4.52; p<0.05] (Table 4).

**Table 4:** Endocrine measurements.

		Corticosterone		Leptin	
		Young Adult	Adult	Young Adult	Adult
**Males**	Vh-Sal	189.4±49.5 (10)	363.2±50.8 (10)	5.2±0.6 (8)	13.2±2.1 (7)
	Vh-MDMA	127.9±36.0 (8)	259.8±53.3^g^ (8)	4.2±0.5 (6)	14.3±2.5 (7)
	THC-Sal	155. 8±43.5 (10)	333.3±33.9 (10)	4.1±0.6 (8)	12.7±1.5 (9)
	THC-MDMA	178.7±31.4 (8)	210.4±50.8^g^ (8)	4.4±1.4 (6)	13.1±1.0 (6)
**Females**	Vh-Sal	415.9±69.8^a^ (10)	795.8±112.4^a^ (10)	6.0±0.9 (9)	8.6±1.0^a^ (10)
	Vh-MDMA	476.1±48.1^a^ (8)	749.6±144.8^a^ (8)	4.5±0.7 (8)	7.7±1.2^a^ (8)
	THC-Sal	446.2±58.7^a^ (10)	659.7±62.9^a^ (10)	4.2±0.8^e^ (9)	6.9±1.5^a^ (9)
	THC-MDMA	454.9±42.1^a^ (6)	684.0±79.1^a^ (6)	2.9±0.7^e^ (6)	7.2±1.6^a^ (5)

Plasma corticosterone and leptin levels (mean ± SEM, ng/ml), in young adolescent (pnd 50) or adult animals (sacrificed around pnd 90). Animals were exposed to increasing doses of THC (2.5, 5 and 10 mg/kg) or vehicle from pnd 28 to 45, and to MDMA (10 mg/kg) or saline twice daily every 5 days from pnd 30 to 45 (see text for details). In parenthesis, the number of animals per experimental group. ANOVA (P<0.05),

a significant overall effect of sex;

e significant overall effect of THC within the same sex group;

g significant MDMA effect within the same sex group.

#### Plasma leptin

Circulating leptin levels were also analyzed at two different time points, in adult (around pnd 90) and in younger rats (pnd 50). In adult animals, there were clear sex differences in plasma leptin levels [F(1,53) = 25.56, p<0.001] in the absence of any drug-induced effect, with males showing higher leptin levels than the corresponding females. By contrast, no effects of sex were observed among the younger animals (pnd 50), although at this age the statistical analysis revealed a residual significant effect of chronic THC administration [F(1,52) = 3.36, p = 0.073]. Indeed, THC induced a significant decrease in leptin levels in females alone (p = 0.05: Table 4).

### Protein expression in the frontal cortex and hippocampus

#### Activity-regulated cytoskeletal-associated protein (Arc)

**Figure 7:**
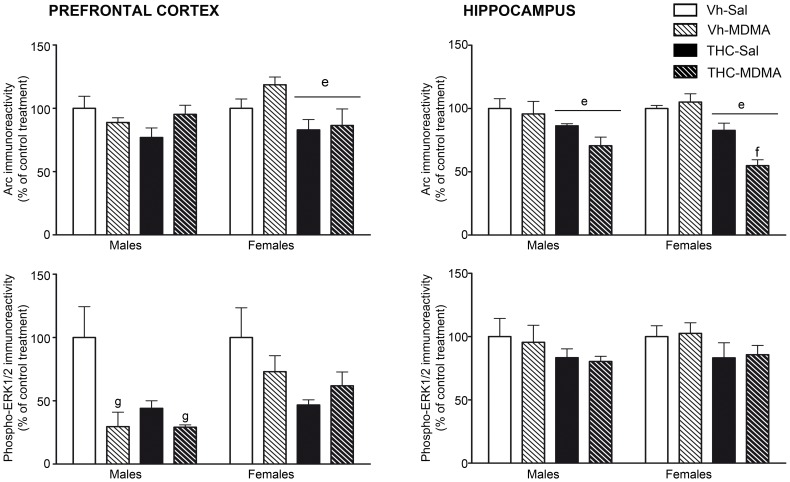
Accumulation of activity-regulated cytoskeletal-associated protein (Arc, upper panels) and phosphorylated extracellular signal-regulated kinase (phosphor-ERK1/2, lower panels) in the frontal cortex (left panels) and hippocampus (right panels) of adult animals sacrificed around pnd 90. Animals were exposed to increasing doses of THC (2.5, 5 and 10 mg/kg) or vehicle from pnd 28 to 45, and to MDMA (10 mg/kg) or saline twice daily every 5 days from pnd 30 to 45 (see text for details). The histograms (mean ± S.E.M.) show the relative change in optical density with respect to the male or female control group. Number of animals per experimental group (n = 4–5). According to ANOVA (P<0.05): (e) significant overall effect of THC within the same sex group; (f) significant MDMA effect among THC-treated animals; (g) significant MDMA effect within the same sex group.

Arc immunoreactivity was analyzed independently in male and female animals. While no significant differences in Arc expression were observed in the prefrontal cortex of male animals, THC administration produced a significant overall decrease in Arc protein in the prefrontal cortex of THC-treated females [F(1,14) = 7.67; p<0.05]. Within the hippocampus, THC administration produced a significant overall decrease in Arc protein in both adult males [F(1,14) = 7.81; p<0.05] and females [F(1,14) = 46.38; p<0.001]. In addition, there was a significant overall effect of MDMA treatment [F(1,14) = 5.31; p<0.05], as well as a significant interaction between THC and MDMA [F(1,14) = 11.08; p<0.01]. Indeed, MDMA exposure exclusively reduced Arc immunoreactivity in THC-treated females [F(1,7) = 13.51; p<0.01], with this drug producing no effect of this drug in animals administered MDMA and the vehicle for THC alone (Fig. 7, upper panels).

#### Phospho-ERK ½

Phospho ERK1/2 was also analyzed separately in male and female animals and there was a large significant overall reduction in phospho ERK1/2 in the prefrontal cortex of adult males following adolescent intermittent MDMA administration [F(1,13) = 19.14; p<0.01] (Fig. 7, lower left panel). No changes in phospho ERK1/2 immunoreactivity were observed in the prefrontal cortex of females or in the hippocampus of either sex (Fig. 7, lower panels).

### Hypothalamic prepro-orexin mRNA expression

In separate two-way ANOVAs performed according to sex failed no significant difference in hypothalamic prepro-orexin mRNA expression was observed in female rats, while there was a significant THC and MDMA treatment interaction [F(1,17) = 5.07; p<0.05] among males. THC administration decreased the amount of prepro-orexin mRNA in the hypothalamus of animals [F(1,9) = 12.17; p<0.01], whereas such an effect was no longer evident in those rats that also received MDMA (Table 5).

**Table 5:** Hypothalamic expression of prepro-orexin transcripts.

	Males	Females
Vh-Sal	1.00±0.22 (6)	1.00±0.31 (6)
Vh-MDMA	0.56±0.29 (5)	0.66±0.15 (3)
THC-Sal	0.12±0.07^h^ (5)	0.68±0.23 (5)
THC-MDMA	0.87±0.38 (5)	1.17±0.39 (6)

Quantitative real-time PCR analysis of hypothalamic preproorexin expression in adult animals sacrificed around pnd 90. Animals were exposed to increasing doses of THC (2.5, 5 and 10 mg/kg or vehicle) from pnd 28 to 45, and to MDMA (10 mg/kg or saline) twice a day every 5 days from pnd 30 to 45 (see text for details). Levels of prepro-orexin mRNA (mean ± S.E.M.) are expressed in arbitrary units in reference to the control animals (Vh-Sal) of their corresponding sex group. In parenthesis, the number of animals per experimental group. ANOVA (P<0.05):

h significant effect of THC in saline-treated male animals.

## Discussion

Cannabis and MDMA are among the most common illicit substances consumed by adolescents, yet the outcomes of the concurrent consumption of both substances remain unclear. Whereas a recent study reported behavioral effects of this combination in male rats [27], to the best of our knowledge this is the first study to analyze sexual dimorphisms directly and adopt a multidisciplinary assessment from molecular to behavioral, including an endocrine and metabolic analyses of the effects of combining these two drugs.

### Body weight and temperature

In this study, THC administration during adolescence decreased the body weight gain in rats [15], [34] and this effect appeared earlier in females than males. This effect may be related to the typical reduction of food intake observed after chronic cannabinoid administration [17], which might be secondary to the down-regulation of CB1 receptors observed in several brain regions, including the hypothalamus [50]. MDMA also reduced body weight gain during drug treatment, both in males and females, in agreement with previous studies [51]. MDMA treatment during adolescence has been shown to impair growth in rats [6] and mice [52], probably due to the anorectic effects of the drug and to the water loss by evaporation resulting from enhanced respiration rates [35]. The rate of body weight gain was lowest in male and female adolescent animals treated with the combination of chronic THC and intermittent MDMA throughout the drug administration period, suggesting that the effect of these drugs may be additive. However, this possibility was not reported in a previous study performed on adolescent male rats [27]. In addition, significant long-term effects were still observed after drug cessation, although these long-term effects were sex dependent since they persisted longer in male rats which still displayed a decreased body weight gain on pnd 70 after THC treatment. Similarly, adolescent MDMA treatment persistently reduced body weight in male animals until pnd 65, whereas this treatment only decreased body weight gain over a shorter period (pnd 50) in females.

As expected, MDMA caused hyperthermia on the first day of injection in both male and female adolescent animals (pnd 30), consistent with previous studies performed on adult [53] and adolescent rats [27]. By contrast, THC administration alone did not modify body temperature. Although THC has been reported to induce hypothermia in rodents, the effects of cannabinoids on body temperature are quite variable and may depend on the dose used and the ambient temperature, among other factors [27], [54]. Interestingly, THC attenuated MDMA-induced hyperthermia in females during the first day of injection. Indeed, concurrent administration of either THC or the synthetic cannabinoid agonist CP 55,940 has been seen to prevent MDMA-induced hyperthermia [26], [27]. In the present study these interactions were further analyzed after chronic treatment. While MDMA-induced hyperthermia did not develop tolerance over days, THC only counteracted the hyperthermic effect of MDMA on the first day of injection. Therefore, this putative protective effect of THC on MDMA-induced hyperthermia may not be representative of current human habits of consumption, which would be more closely modeled by chronic studies. Moreover, species differences could be important since THC did not attenuate MDMA-induced hyperthermia in humans but rather, it may even enhance it [55].

### Behavioral outcomes

General activity in the holeboard was not modified by the different drug treatments. However, THC decreased the distance traveled in the interior of the arena, while hole-directed explorative behavior was modified by MDMA. In fact, both the frequency and the time spent exploring the holes were decreased by MDMA, in agreement with previous results [27]. These data suggest that one day after the pharmacological treatment both drugs increased emotionality or anxiety in this model. By contrast, MDMA administration increased the open-arm exploration in the EPM, which can be interpreted as an anxiolytic-like effect [36] or as an increase in risk-taking behavior [15], [56]. The fact that MDMA-treated animals fell more often from the EPM suggests enhanced risk taking and impulsivity. Indeed, previous data suggests that intermittent adolescent MDMA exposure causes increased impulsivity in the EPM [27], [35], consistent with the results obtained in ecstasy users [57], [58]. Both, THC and MDMA given alone decreased the time spent exploring the central region of the open field, which further supports increased emotionality/anxiety and the possible enhancement of impulsivity in the EPM. Accordingly, adolescent MDMA administration in rats [51] and humans [59] may lead to increased anxiety in adulthood. Sex differences were revealed in these tests since adolescent female rats explored the open arms of the EPM for longer than males and they displayed greater motor activity in the open field, consistent with our previous findings [16].

In the NOT, THC administration during adolescence affected memory in adult female animals, suggesting an enhanced vulnerability of this sex to the long-term effects of THC on certain emotional and cognitive responses. Accordingly, chronic THC consumption by adolescent rats induces depressive-like behavior in adult female but not male rats [34]. The peripubertal period appears to be critical for the development of the endocannabinoid system and chronic cannabinoid exposure during this period may lead to persistent functional impairment of the endocannabinoid system [19], [20], [28]. Indeed, chronic cannabinoid administration during the periadolescent period induces persistent memory impairment in adult rats [16], [18]. Moreover, chronic THC administration produces a long-lasting impairment of the spatial working memory in adult rats [60], although this alteration may not be produced with lower doses of THC [61].

The greater sensitivity of female adolescents to the deleterious effects of cannabinoid exposure is evident in both humans and rodents [62]. These sex differences might be related to baseline sex differences in the density and activity of brain CB1 receptors. Indeed, female rats display higher CB1 receptor-mediated G protein activation in the hippocampal formation than males [16], probably to compensate for the lower density of CB1 receptors in this brain area in females [63]. Gonadal hormones inducing brain sexual differentiation during perinatal and periadolescent periods, particularly estradiol, seems to be critically involved in the sexually dimorphic effects of cannabinoids in adults (see [20] and [62] for review). Pharmacokinetic factors may also be involved in these differences in response to cannabinoids since the cytochrome P450 responsible for cannabinoid metabolism appears to be sex-specific in rats, as witnessed by the preferential metabolism of THC to its highly active metabolite in females but not in males. In addition, THC is stored in fatty tissue that tends to be more prevalent in females than males, which may also influence the distribution and excretion of THC. These differences in metabolism may contribute to the more pronounced effects of THC in female rodents and humans [64].

The early adolescent period appears to be associated with a unique vulnerability to some of the adverse effects of cannabinoids. Thus, acute and chronic administration of the cannabinoid agonist WIN55,212-2 induced more severe cognitive and social behavioral disturbances in pubertal than in adult rats [65]. These long-term cognitive effects of adolescent cannabinoid exposure seem to be related to impaired synaptic function in the hippocampus [60], [63]. Indeed, early-onset cannabis users exhibit poorer cognitive performance than late-onset users or control subjects [66]. Under the present experimental conditions, MDMA did not modify long-term cognitive responses in the NOT, in agreement with previous studies [51]. However, MDMA may be detrimental to cognition in other paradigms of working memory in rats [67], as well as to working memory [68] and associative learning [69] in current and abstinent human users. It has been suggested that a critical period may exist between pnd 11–20 for the long-term effects of MDMA on cognition [70]. The results presented here also indicate that general object exploration during the test session was impaired by MDMA treatment, which may suggest a poorer attention capability, consistent with the results obtained in the PPI test.

Prepulse inhibition of the acoustic startle response has been widely used as a measure of sensorimotor gating and deficient PPI has been reported in several neuropsychiatric disorders [38]. Drugs that release serotonin, such as MDMA, acutely impair sensorimotor gating [71] in a dose-dependent manner in both sexes [32], although no effect of MDMA was reported after repeated intermittent administration [72]. The present results show that adolescent MDMA administration decreased PPI performance in adulthood. Therefore, adolescent animals seem to be particularly vulnerable to the effects of MDMA on PPI considering the negative results obtained with this administration schedule in adult rats [72], although different experimental conditions were used in these two studies. THC did not modify PPI in our experimental conditions, in contrast to previous reports showing PPI impairment in adult females after adolescent treatment with CP 55,940 [15], as well as in males after peripubertal exposure to WIN55,212-2 [17], [73]. Thus, the doses of THC used here may not have been sufficiently high to impair PPI. At a prepulse intensity of 75dB, MDMA was only effective in reducing PPI when combined with THC treatment, suggesting that THC may enhance the effects of MDMA and that the combination of both drugs may increase the possibility of developing psychiatric symptoms. An impaired PPI is observed in patients suffering from psychotic symptoms and personality traits that may enhance drug consumption [74]. Therefore, adolescent MDMA exposure may increase vulnerability to develop psychiatric disorders and facilitate the co-abuse of other drugs.

### Hormone levels

In agreement with previous studies using cannabinoid agonists [15], [17], chronic THC administration during adolescence did not affect basal corticosterone levels in either sex. By contrast, adolescent intermittent MDMA exposure induced a long-term decrease in corticosterone levels in male rats. Previous studies reported that acute MDMA treatment produced a short-term dose-dependent increase in corticosterone levels [75], and this effect produced partial tolerance after two weeks of intermittent treatment [76]. A different protocol of daily THC administration and intermittent MDMA treatment every fifth day from postnatal day 35 to 60 did not provoke any effect on basal corticosterone levels or the corticosterone stress response [27]. These differences with the results of the present study might be due to the different experimental conditions. By contrast, elevated baseline cortisol and ACTH levels were revealed in experienced human ecstasy users compared to non-using controls [77], suggesting species differences in neuroendocrine reactivity to MDMA. As for the majority of the parameters studied, corticosterone levels showed a marked sexual dimorphism among control animals, with higher circulating hormone levels in females than in males during adolescence and adulthood, consistent with previous data from adult [17] and adolescent [47] rats.

Circulating leptin levels were not significantly modified by THC or MDMA, although adolescent THC administration tended to decrease leptin levels in female animals consistent with the reduced leptin levels revealed in males after chronic adolescent treatment with the cannabinoid agonist CP 55,940 [15]. These results suggest that exposure to cannabinoids during the periadolescent period may reduce circulating leptin levels, although the extent of this effect may depend on the specific cannabinoid agonist, dose and administration schedule used.

Previous studies suggested that acute MDMA administration induced a transient dose-dependent effect on serum leptin levels [45], and this may explain the absence of changes in leptin levels after a long wash-out period. Sex differences were also revealed among adult control animals, with males showing higher leptin levels than the corresponding females, although no sex differences were observed in young animals, in accordance with previous studies showing higher plasma leptin levels in males than in females only after puberty [78].

### Neurochemical changes

Several neurochemical responses were evaluated to identify possible neurobiological mechanisms related to the behavioral changes induced in adult animals by chronic exposure to THC and/or MDMA. Arc immunoreactivity was first investigated, which is protein rapidly modulated in a synaptic activity-dependent manner [79], [80]. In the prefrontal cortex, no significant differences in Arc were observed in male animals, while a decrease was evident in THC-treated females. In the hippocampus, adolescent THC administration decreased the accumulation of Arc protien in adult males and females. In addition, an interaction between both drugs was found in this brain structure since MDMA exclusively reduced Arc immunoreactivity in THC-treated females. Interestingly, local down-regulation of Arc protein in the hippocampus using antisense oligodeoxynucleotides selectively impairs the consolidation of spatial learning [81], pointing to a possible relationship between the memory deficits found in the female rats administered THC and MDMA, and the decrease in Arc expression.

We also investigated phospho-ERK 1/2 immunoreactivity, which has been reported elsewhere to be modulated by THC [39] and MDMA [40]. Changes in ERK1/2 phosphorylation have been associated with mood changes in animal models [41] and in our experimental setting, ERK1/2 signaling was down-regulated in the frontal cortex of adult male rats exposed to MDMA during the adolescence, although no significant effects were observed in females. In MDMA treated males, ERK 1/2 changes may be related to the behavioral alterations found immediately after the end of the pharmacological treatment in the holeboard test (i.e.: a decrease in the frequency and time spent head-dipping, indicative of increased emotionality) and in the EPM, where they showed increased risk taking behavior. However, MDMA females showed similar short-term behavioral alterations in the holeboard test and in the EPM but no changes in their ERK1/2 expression at adulthood. It is known that estradiol exerts some of its neuroprotective effects by activating ERK signaling [82]. It is tempting to speculate that in females, this gonadal hormone restores this signaling system during the wash-out period between the pharmacological treatments administered here and the sample collection. In our hands, THC did not induce any significant effect on ERK1/2 signaling despite the fact that immediately after acute and chronic treatment with THC, an increase in phospho-ERK immunoreactivity has been described in different brain areas [39]. However, in our protocol the THC treatment ended long before the biochemical assays were performed and therefore, it is likely that ERK1/2 signaling has been totally restored by then. In other words, the failure to detect a correlation in the present study may be due to the time that past between the behavioral and biochemical evaluations.

Lastly, THC induced a specific decrease in hypothalamic prepro-orexin expression in male animals. Given that CB1R axonal immunoreactivity has been reported in the medial hypothalamus [83], the action of THC on hypothalamic CB1 receptors may have induced the down-regulation in preproorexin. Indeed, a recent study has demonstrated that CB1 receptor agonists modulate the activity of orexin/hypocretin neurons, probably by interacting with presynaptic CB1 receptors located on GABA terminals [84]. Moreover, the specific down-regulation of hypothalamic prepro-orexin found in adult males exposed to THC alone was prevented when these rats received both THC and MDMA during adolescence. Interestingly, repeated administration of another psychostimulant, cocaine, induces long-lasting potentiation of glutamatergic synapses on orexin neurons in mice [85]. A similar excitatory mechanism could explain the blockade of the effects induced by THC treatment on orexin expression when combined with intermittent MDMA treatment. No effects were found in females for prepro-orexin mRNA levels, which again suggests a neuroprotective mechanism of hypothalamic orexin neurons against THC-related impairments in these mice, probably in connection with sexual steroids.

## Conclusions

It has been suggested that acute THC and MDMA administration may have opposing effects on animals, although their negative effects might be additive when taken chronically [22]. The animal model used in this study mimics the current habits of human adolescent consumption and thus, it may provide a more reliable picture of the detrimental consequences of polydrug abuse than acute experiments, particularly regarding THC and MDMA that are frequently co-abused among adolescents. Whereas there may be some apparent balancing of the detrimental effects of one of the two drugs during an initial exposure, such as in terms of body temperature, the opposite seem to occur in the long-term after repeated exposure, as seen here for body weight and the PPI response. The reported behavioral effects of THC and/or MDMA are possibly related to alterations in neurotransmitter systems that have already been shown to be altered after consumption of these drugs. For instance, a depletion of serotorinergic nerve terminals in diverse brain regions may be produced by MDMA administration and this may underlie the increase in emotionality and risk-taking behavior, as well as the weight loss, reported immediately after drug cessation. In turn, the persistent changes in the endocannabinoid system induced by chronic THC administration may be related to deficits in working memory evident in female animals, and/or with the impairments to PPI responses. Further studies will be required to unravel the molecular mechanisms underlying the long-term behavioral effects of both drugs.

Despite the experimental limitations, such as the application of only one schedule of drug administration, the use of only one dose of MDMA and increasing doses of THC, the selection of this animal model to mimic the current habits of human adolescent consumption opens new avenues for translational research in the field of drug consumption. In addition, the study of endocrine and neurochemical data in the same animal at different time points allows us to establish potential associations, although more additional experiments will be necessary to obtain evidence for causal relationships. The data presented revealed diverse sexual dimorphisms that are probably due to the organizational effects of perinatal gonadal hormones during a critical period of brain sexual differentiation [86], and/or to their organizational effects and the activation they produce during the periadolescent period [20], [28]. A better understanding of the mechanisms underlying the sexually dimorphic effects of cannabinoids [20], [28] and MDMA [87] will facilitate the development of sex-specific preventive and therapeutic strategies.
