# Correction: Sex-Dependent Psychoneuroendocrine Effects of THC and MDMA in an Animal Model of Adolescent Drug Consumption

**DOI:** 10.1371/annotation/89e38eb6-e8e3-402d-a6fb-84ebf9fcbb27

**Published:** 2013-12-16

**Authors:** Alvaro Llorente-Berzal, Emma Puighermanal, Aurelijus Burokas, Andrés Ozaita, Rafael Maldonado, Eva M. Marco, Maria-Paz Viveros

There is an error in the "Immunoblotting of the frontal cortex and hippocampus" section under "Materials and Methods." The correct paragraph for this section is:

Protein samples were prepared as described previously [49] and they were analyzed in western blots probed with antibodies against ERK1/2 and phospho-ERK1/2 (Thr202/ Tyr204/ Thr185/ Tyr187: Sigma, Madrid, Spain), against Arc (Santa Cruz Biotechnologies, Santa Cruz, CA, USA) and against glyceraldehyde 3- phosphate dehydrogenase (GAPDH: Santa Cruz Biotechnologies, Santa Cruz, CA, USA) to ensure accurate protein loading. Optical densities of relevant immunoreactive bands were quantified after acquisition on a ChemiDoc XRS System (Bio-Rad, Hercules, CA, USA) controlled by the Quantity One software version 4.6.3 (Bio-Rad, Hercules, CA, USA). The data were expressed as the relative change in optical density with respect to the male or female control group, considered as 100%. Since samples from male and female animals were analyzed in separate blots, sex-dependent effects could not be analyzed for these measurements. 

